# Quantitative measurements of α-synuclein seeds in CSF inform diagnosis of synucleinopathies

**DOI:** 10.1177/1877718X251379292

**Published:** 2025-10-03

**Authors:** Ilham Y Abdi, Indulekha P Sudhakaran, Simona S Ghanem, Nishant N Vaikath, Nour Majbour, Yee Y Goh, Nirosen Vijiaratnam, Christine Girges, Vasilios C Constantinides, Elisabeth Kapaki, George P Paraskevas, Sandrina Weber, Gholam Adeli, Kostas Vekrellis, Daniel Erskine, Michele Hu, Thomas Foltynie, Henry Houlden, Laura Parkkinen, Wilma DJ van de Berg, Brit Mollenhauer, Michael G Schlossmacher, Omar MA El-Agnaf

**Affiliations:** 1 Neurological Disorder Research Center, Qatar Biomedical Research Institute, Hamad Bin Khalifa University, Qatar Foundation, Doha, Qatar; 2 College of Health and Life Sciences, Hamad Bin Khalifa University, Qatar Foundation, Doha, Qatar; 3 Translational Medicine, Neuroscience, Pharmaceuticals R&D, AstraZeneca, Cambridge, UK; 4 Department of Neuromuscular Diseases, Neuromuscular Diseases, UCL Queen Square Institute of Neurology, London, UK; 5 Department of Clinical and Movement Neurosciences, UCL Queen Square Institute of Neurology, London, UK; 6 Movement Disorders Centre, UCL Queen Square Institute of Neurology, London, UK; 7 Neurochemistry and Biomarkers Unit, 1st Department of Neurology, National and Kapodistrian University of Athens, Athens, Greece; 8 Ward of Cognitive and movement Disorders, 1st Department of Neurology, National and Kapodistrian University of Athens, Athens, Greece; 9 Department of Neurology, University Medical Center Göttingen, Göttingen, Germany; 10 Paracelsus-Elena-Klinik, Kassel, Germany; 11 Neuroscience Institute, Hamad Medical Corporation, Doha, Qatar; 12 Center of Basic Research, Biomedical Research Foundation of the Academy of Athens, Athens, Greece; 13 Translational and Clinical Research Institute, Newcastle University, Newcastle, UK; 14 Nuffield Department of Clinical Neurosciences, Oxford Parkinson's Disease Centre, University of Oxford, Oxford, UK; 15 Department of Anatomy and Neurosciences, Amsterdam UMC Vrije University Amsterdam, Amsterdam, Netherlands; 16 Neuroscience Program, The Ottawa Hospital, Ottawa, Ontario, Canada; 17 University of Ottawa Brain and Mind Research Institute, Ottawa, Ontario, Canada; 18 Division of Neurology, Department of Medicine, The Ottawa Hospital, Ottawa, Ontario, Canada

**Keywords:** α-synuclein, synucleinopathies, biomarkers, immunoassays, SAA

## Abstract

Diagnosing α-synucleinopathies and assessing target engagement in trials is hindered by the lack of reliable biomarkers. Here, we introduce a first-in-kind quantitative, highly sensitive, and disease-specific diagnostic assay, named Seeding Amplification ImmunoAssay (SAIA), developed and validated to detect synucleinopathy-linked disorders. To this end, we used wide range of specimens, including 38 brain homogenates (BH) and 559 cerebrospinal fluid (CSF) samples from subjects with diverse synucleinopathy disorders, non-synucleinopathy diseases, idiopathic REM sleep behavior disorder (iRBD), and controls. SAIA generated robust results detecting disease-related α-synuclein seeds in BH samples at attogram levels, as referenced to preformed fibrils of α-synuclein. Furthermore, we conducted side-by-side comparisons between SAIA and a traditional Seeding Amplification Assay (SAA), which revealed high concordance. Further, SAIA distinguished synucleinopathies from non-synucleinopathies and controls with sensitivities and specificities ranging between 80–100% and area under the curve values exceeding 0.9. SAIA also accurately identified 24/24 (100%) iRBD cases, considered a prodromal state of PD, with 100% sensitivity and 80% specificity. Further optimization of SAIA through timepoint analyses revealed that shorter incubation times enhanced the assay's specificity for distinguishing MSA from PD highlighting the potential for improved differentiation between specific synucleinopathies. In conclusion, SAIA represents a novel, high-throughput method to screen, diagnose, and monitor synucleinopathy disorders in living subjects, offering significant improvements over existing methods through its quantitative output, shorter incubation time, and simplified workflow, features that enhance its suitability for clinical trial applications.

## Plain language summary

A new sensitive test to detect early Parkinson's and related brain disorders using brain and spinal fluid samples

Parkinson's disease and related conditions, known as synucleinopathies, are caused by the buildup of a protein called alpha-synuclein in the brain. Diagnosing these conditions early and accurately is a major challenge, especially before obvious symptoms appear. It is also difficult to track how well treatments work in clinical trials because current tests are not sensitive or specific enough. In this study, researchers developed a new laboratory test called the Seeding Amplification ImmunoAssay (SAIA). This test can detect very small amounts of abnormal alpha-synuclein proteins in samples taken from the brain and spinal fluid (cerebrospinal fluid or CSF). The test was designed to be both highly sensitive and specific to diseases like Parkinson's disease, dementia with Lewy bodies, and multiple system atrophy. The team tested SAIA on a wide range of samples from people with different brain conditions, including 38 brain tissue samples and 559 CSF samples. These came from individuals with synucleinopathies, nonrelated brain conditions, a sleep disorder called iRBD (which can be an early sign of Parkinson's), and healthy individuals. The new test successfully detected disease-linked alpha-synuclein proteins, even in extremely small amounts. Importantly, SAIA was able to clearly distinguish people with synucleinopathies from those without, and it identified all 24 iRBD cases correctly, showing promise for detecting disease even before major symptoms appear. The researchers also found that testing the samples for a shorter time improved the ability to tell different types of synucleinopathies apart, such as Parkinson's versus multiple system atrophy. In summary, SAIA is a promising tool for improving early diagnosis, helping select the right patients for clinical trials, and tracking how well new treatments are working.

## Background

Synucleinopathies, including Parkinson's disease (PD), dementia with Lewy bodies (DLB), Parkinson's disease dementia (PDD), and multiple system atrophy (MSA), are associated with the misfolding and aggregation of the protein α-synuclein into toxic insoluble fibrils in the selectively vulnerable regions of the brain.^1^ Despite the tangible progress made in our understanding of disease, there's unmet need for establishing reliable and disease-specific biomarkers for diagnosis or progression of synucleinopathies.^2,3^ This need can only be achieved through developing innovative and robust methods and assays for biomarkers discovery and assessments.

Various approaches have been employed to detect misfolded α-synuclein aggregates in tissues and biological samples. Among them are antibody-based immunoassays like ELISA,^4–7^ as well as assays that exploit the self-propagating property of α-synuclein aggregates, such as seed amplification assay (SAA).^8,9^ Both approaches have demonstrated notable advantages in the detection and analysis of synucleinopathies in living subjects.^6,10–14^ Seeding assays, in particular, demonstrate high sensitivity and specificity in detecting α-synuclein aggregates at low levels and even at early stages of the disease, enabling early diagnosis.^15^ Several of these studies have shown remarkable specificity scores, effectively discriminating PD, DLB, MSA patients from non-synucleinopathies cases and enhancing our understanding of disease molecular diagnosis.^16–26^ On the other hand, ELISA platforms provide a versatile and widely used approach for quantifying α-synuclein protein levels and/or modified metabolites thereof.

Despite their effectiveness, both SAA and ELISA techniques have limitations. SAA despite its sensitivity and promising diagnostic potential lacks direct quantification capabilities, thus meaning it is limited to a binary outcome of positive or negative, and lacking additional data to support stratification of cases for prognostics or clinical trials. Furthermore, SAA kinetic parameters are not always reproducible across cohorts or protocols, which limits their reliability for quantitative interpretation. Prior attempts to relate SAA readouts to clinical measures have relied on kinetic metrics, with mixed reproducibility across studies.^16,26–33^ These methods necessitate specialized technical expertise and well-equipped laboratories, making them less accessible in routine clinical settings.^34^ On the other hand, ELISAs, although widely used, have limited sensitivity in detecting low levels of pathological α-synuclein species in biological samples.^19,24,35,36^ Limitations in these techniques, albeit being the most advanced diagnostic techniques currently available, highlight a need for improvements that can allow for a significant advancement in clinical diagnostics of synucleinopathies.

**Figure 1.**
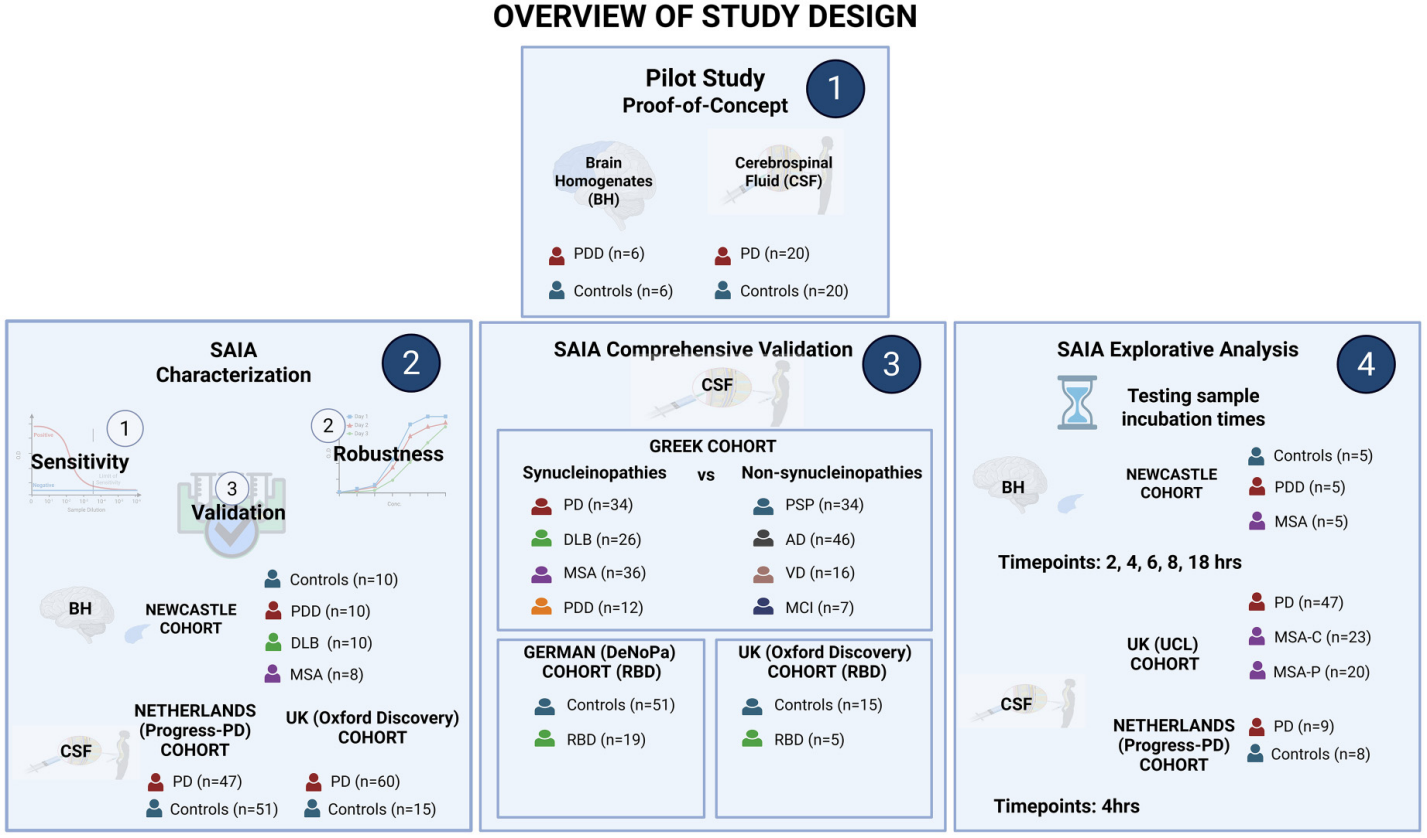
Overview of study design. *Schematic presentation of study design. 1) Proof of concept was established in a small number of BH and CSF samples. 2) Assay was characterized by its sensitivity, robustness and validated in larger number of BH and CSF samples. 3) Assay was further validated in a larger comprehensive set of clinical well-described cohorts. 4) Explorative experiments to improve assay specificity were conducted by testing different sample incubation times (2, 4, 6, 8, and 18hrs) in BH samples followed by validation of the optimal incubation time (4hrs) in CSF samples *(Created with Biorender.com).

With this in mind, we aimed to develop a novel immunoassay that not only amplified disease-associated α-synuclein seeds in samples but also quantified them within the same assay, which we’ve termed “seeding amplification immunoassay (SAIA)”. Herein we present proof-of-concept of the SAIA technique, its development as a high throughput platform, and our validation efforts regarding its application to diverse sample types, cohorts and distinct clinical conditions, thereby offering valuable insights into the diagnostic capabilities of the assay (Figure 1). The results underscore the assay's effectiveness in detecting α-synuclein seeding activity in various biological matrices, that include 38 BH specimens and 559 CSF samples. Our findings highlight the versatile and diagnostic value of SAIA in different cohorts that include synucleinopathies and prodromal cases.

## Materials and methods

### Experimental design

**Figure 2.**
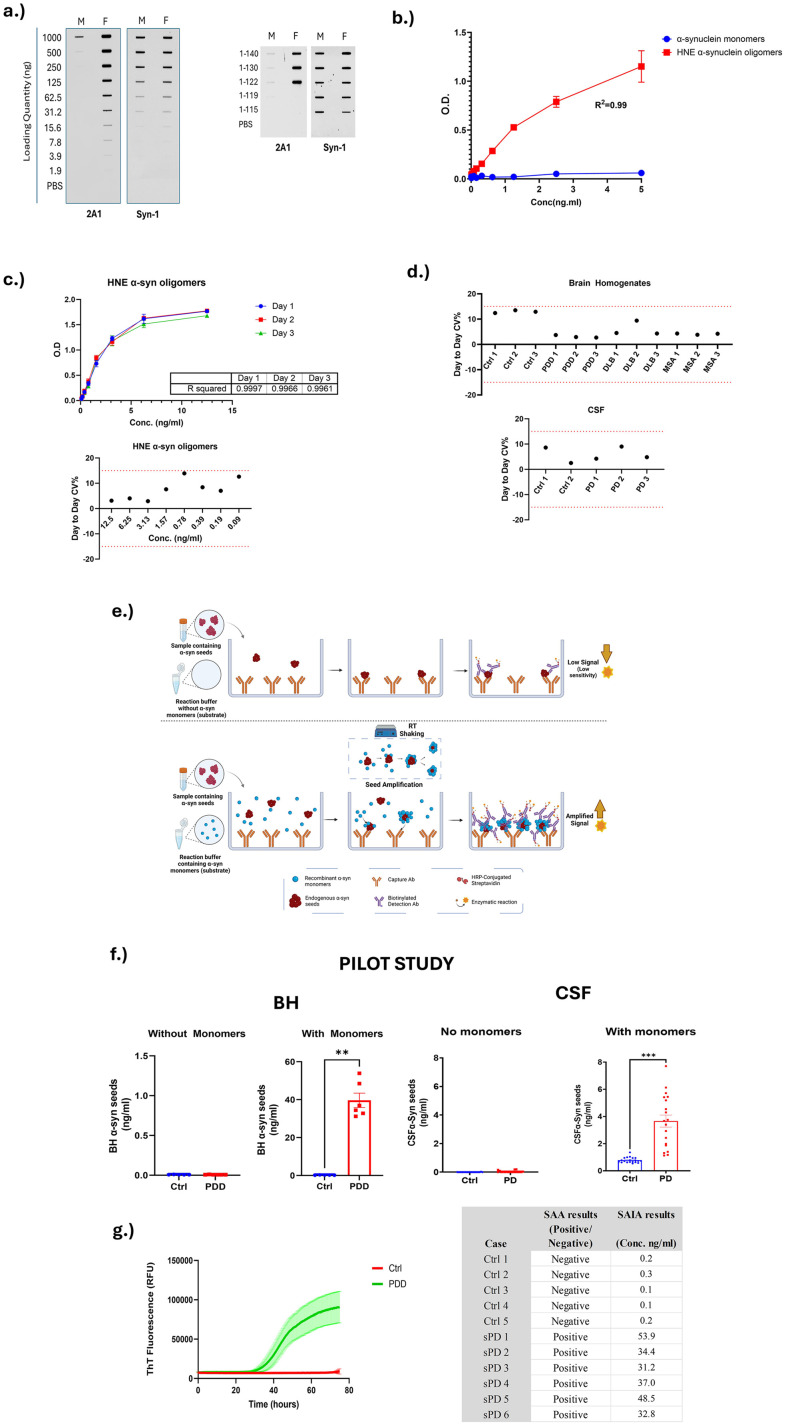
Illustration of seeding amplification immunoassay concept, assay characterization and proof of concept- pilot study with BH and CSF. (a) Slot-blot assay showing our conformation-specific anti-α-synuclein mAb 2A1 preferentially detects α-synuclein aggregates, while Syn-1 binds both monomers and aggregates. 2A1 also recognizes full-length and truncated α-synuclein down to residue 122, whereas Syn-1 detects all fragments. (b) sELISA assay using 2A1 Ab showing selective detection of HNE-modified α-synuclein oligomers (red squares) compared to α-synuclein monomers (blue circles). A strong linear response (R² = 0.99) was observed for oligomers, while monomers showed minimal signal, confirming assay specificity for aggregated species. Data are presented as mean ± SD. (c) Inter-assay variability plots of HNE α-synuclein oligomer standards tested over three non-consecutive days. (d) BH and CSF samples tested over two non-consecutive days. (e) Diagrammatic representation of SAIA concept. The top panel of the diagram presents a conventional sandwich ELISA protocol employing without the seeding amplification step. In this setup, diluted samples are processed in a reaction buffer without adding recombinant α-synuclein monomer to serve as a substrate. Although this method allows for the capture and detection of endogenous α-syn seeds within the samples, it achieves this with limited sensitivity. The bottom panel illustrates an enhancement to this method under identical experimental conditions (same reaction buffer, shaking, and temperature). By introducing α-syn monomers, there is an amplification of endogenous α-syn seeds. This amplification could occur through two possible mechanisms: either directly in solution, leading to the formation of a complex aggregate of recombinant and endogenous alpha-synuclein captured by the monoclonal antibody, or by the initial capture of endogenous seeds by the antibody followed by seeding. Such amplification significantly increases the number of potential binding sites available for the detection antibody, biotinylated 2A1, thereby substantially enhancing the sensitivity and the overall signal detected in the assay. (Created with BioRender.com). (f) Seeding effect, indicated by the increase in measured seeded α-synuclein, is evident in pilot study of PD BH and CSF samples respectively, in the presence of the substrate, α-synuclein monomers, whereas controls maintained a low signal. The error bars represent the standard error of the mean. (g) SAA reactions of BH samples were performed in triplicate from both control individuals (red) and patients with PDD (green). The solid line denotes the mean ThT signal across triplicate wells, while the colored ribbon signifies the standard error.

Our experimental approach can be summarized in three main steps: 1.) “Seed Capture”- biofluid samples, containing α-synuclein seeds, are subjected to capture by the conformation-specific anti-α-synuclein antibody, 2.) “Seed Amplification”- simultaneously, the captured seeds are seeded by recombinant α-synuclein monomers present in a reaction mix promoted by optimal incubation and shaking conditions, and lastly 3.) “Seed Detection”- The amplified seeds are then detected with an anti-α-synuclein antibody, generating a signal that could be extrapolated to a standard curve to determine the concentration of amplified α-synuclein seeds in the samples (Figure 2(a)). This assay setup allows for the capture, amplification, and quantification of α-synuclein seeds exclusively in samples where these seeds were present. The conformation specific anti-α-synuclein Ab used for this study is our in-house produced and characterized proprietary monoclonal Ab 2A1 (QABY Biotech, Qatar).^37^ Prior to public listing, requests can be made directed to the corresponding author; materials will be supplied under an MTA in coordination with QABY.

The proof-of-concept was first conducted in a pilot study using BH and CSF samples from individuals with PD and age-matched controls (n = 6/group for BH, n = 20/group for CSF). All brain samples were taken from the frontal cortex, specifically the superior frontal gyrus. Results were validated through a side-by-side comparison with traditional SAA, with both assays confirming disease-specific α-synuclein seeding activity in PDD and PD samples.

The sensitivity of the assay as explored by testing serial dilutions, 1:100 to 1:204.2k, of BH from PD, DLB, MSA to assess the lowest dilution that can be used to amplified and quantified. Similarly, we also tested spiking dilutions of α-synuclein pre-formed fibrils in sample buffer and BH from control cases for a comparison between the assay's sensitivity to endogenous α-synuclein compared to recombinant pre-formed fibrils.

A larger validation cohort of brain homogenate samples from subjects with PDD (n = 10), DLB (n = 10), and MSA (n = 8), and controls (n = 10) was conducted. We then validated SAIA using CSF cohorts from the UK (Oxford Discovery; PD n = 60 and Ctrl n = 15) and the Netherlands (Progress-PD; PD n = 47 and Ctrl n = 51). We conducted an SAA for the BH samples and used previously published SAA results^26^ of the UK (Oxford Discovery) CSF cohort to compare assay performances between SAA and SAIA. This phase evaluated the assay's diagnostic sensitivity and specificity for PD.

The assay was further evaluated for its performance in CSF samples by validating in other CSF cohorts. The cohorts covered various disease groups to evaluate its 1.) Sensitivity at detecting at risk prodromal cases of synucleinopathies- German (DeNoPa) cohort (RBD vs Ctrl, n = 19 and 51 respectively) and, UK (Oxford Discovery) cohort (RBD n = 5 vs Ctrl n = 15) 2.) specificity to synucleinopathies compared to non-synucleinopathies-Greek cohort (PD, PDD, DLB, and MSA patients (n = 34,12, 26, and 36, respectively), as well as non-synucleinopathy including progressive supranuclear palsy (PSP), Alzheimer's disease (AD), vascular dementia (VD), and mild cognitive impairment (MCI) patients (n = 34, 46, 16, and 7, respectively).

To enhance SAIA specificity for differentiating between synucleinopathies like PD and MSA, timepoint experiments were conducted. BH samples from PD and MSA cases were incubated for varying durations, ranging from 2 to 18 h, to investigate time-dependent aggregation kinetics. Following the BH analysis, CSF samples were tested at selected timepoints to assess the influence of incubation times on specificity. This iterative approach was designed to determine optimal incubation conditions for distinguishing PD and MSA based on aggregation behavior, aiming to refine SAIA's diagnostic capabilities.

Although the assays were performed in a blinded manner, the threshold used to define positivity was determined retrospectively after analyzing all groups. As such, this study was not designed as a prospective diagnostic validation but as an initial exploration of the assay's diagnostic potential. This comprehensive experimental design allowed us to rigorously evaluate the SAIA's performance across a range of sample types and clinical conditions, providing valuable insights into its potential clinical utility in synucleinopathy research and diagnosis.

### Clinical cohorts

#### Post-mortem human brain tissues (Newcastle cohort)

Brain tissue was obtained from Newcastle Brain Tissue Resource, a UK Human Tissue Authority-approved research tissue repository, and ethical approval was granted by the Joint Ethics Committee of Newcastle and North Tyneside Health Authority (ref: 19/NE/0008).

At autopsy, tissue was prepared by removing the brainstem at the level of the red nucleus and the cerebrum hemisected through the corpus callosum. The right hemisphere was immersion-fixed in neutral buffered formalin for subsequent paraffin wax embedding for neuropathological diagnosis, based on standardized neuropathological scales.^38^ The left hemisphere was sliced into 1 cm sections and rapidly frozen at −120°C between copper plates for molecular analysis.

PDD (n = 10) and DLB (n = 10) cases were identified and thus included in the present study on the basis of clinico-pathological diagnosis of either PDD or DLB by review of clinical records and neuropathological post-mortem report by experienced senior Old Age Psychiatrists and Neuropathologists, and on the basis of established international guidelines.^39^ MSA cases were included on the basis of clinical impression of MSA (n = 6 parkinsonian type (MSA-P) and two cerebellar type (MSA-C)) and the presence of α-synuclein-immunoreactive Papp-Lantos bodies/glial cytoplasmic inclusions on neuropathological post-mortem examination.^40^ Control cases were included on the basis of an absence of α-synuclein pathology and did not show any other prominent protein pathology.

#### CSF samples

##### Netherlands (Progress-PD) cohort

CSF samples collected from 47 PD patients and 51 age-matched controls, recruited at the Amsterdam UMC outpatient clinic were included.^41,42^ The study was approved by the local medical ethical committee of VU University Medical Center, Amsterdam. All patients gave written informed consent at study entry for the use of clinical information and CSF material for scientific research purposes. All patients were diagnosed with PD by movement disorder specialists according to the United Kingdom Parkinson's Disease Society Brain Bank clinical diagnostic criteria.^43^ The severity of the motor symptoms was assessed using the UPDRS-III.

##### Greek cohort

All patients were examined from 2011 to 2020 at the ward of Cognitive and Movement Disorders of Eginition Hospital. A total of 212 patients were included for the purposes of this study, of whom 109 had synucleinopathy. These included 41 clinically established PD, six patients with probable PDD, 26 patients with probable DLB, and 36 patients with probable MSA. Additionally, 103 patients with a non-synucleinopathic underlying pathology were included, comprising 34 patients with PSP, 46 patients with probable AD, 16 patients with VaD, and 7 patients with MCI. Diagnoses were set based on established diagnostic criteria.^39,44–50^

##### German (DeNoPa) cohort

In this single-center clinical cohort study, the DeNoPa cohort was utilized, including 51 matched HC and 19 individuals with video-supported polysomnographically (vPSG) verified idiopathic REM sleep behavior disorder (iRBD). Controls were frequency matched based on age, sex, and education, and were required to be between 40 and 85 years old, free from central nervous system conditions, and without a family history of idiopathic PD. The iRBD group was diagnosed clinically and confirmed through vPSG, with exclusion criteria for medication-induced iRBD and severe sleep apnea. Dopamine transporter imaging was performed in iRBD subjects, and iRBD diagnosis was based on video analysis of vPSG data using established criteria. Details of the in-and exclusion criteria for the cohort have been reported in previous publications.

##### UK (Oxford Discovery) cohort

The UK cohort is made up of 60 PD patients, 5 iRBD patients and 15 controls from a large, longitudinal Discovery study at the Oxford Parkinson's Disease Centre.^51^ PD patients were diagnosed using UK PD Brain Bank criteria and iRBD using polysomnographic evidence (ICSD3) by specialist neurologists. Ethical approval was secured from local committees, and all participants signed written informed consent in accordance with the Declaration of Helsinki.

##### UK (UCL) cohort

The cohort of CSF samples provided from the University College of London (UCL) is a made up of baseline CSF samples collected from MSA and PD patients in two studies: 13 MSA patients from the “Progressive Supranuclear Palsy-Corticobasal Syndrome-Multiple System Atrophy” (PROSPECT-M-UK) study^52^ and 30 MSA patients and 57 PD patients from the Exenatide Study.^53^

### Production and purification of α-synuclein

Full length human α-synuclein protein was expressed in E. coli BL21 (DE3) cells and purified as previously described.^37^ Post purification by gel filtration and ion exchange, the final protein fractions were pooled and dialyzed against 1X PBS. Dialyzed proteins were filtered in a 100-kDa MWCO filter (Merck Millipore) and the protein concentrations were quantified using the Pierce BCA protein assay kit (Thermo Fisher). Purified stock protein was stored at −80 °C. For seeding amplification assays, the stock protein is thawed, refiltered and quantified prior to each experiment.

### Filter retardation assay for antibody characterization

Serial dilutions of full-length human α-synuclein monomers and aggregates (ranging from 1000 ng to 0 ng), along with truncated α-synuclein variants (1–107, 1–115, 1–119, 1–122, 1–123, 1–130, 1–135, and 1–140) at 50 ng, were applied to a nitrocellulose membrane using a filtration apparatus. The membrane was probed with 2A1 and Syn-1 antibodies (50 µg/mL; Syn-1 from BD Biosciences), followed by incubation with HRP-conjugated goat anti-mouse secondary antibody (1:20,000 dilution). Detection was performed using SuperSignal™ West Pico PLUS Chemiluminescent Substrate (Thermo Fisher Scientific).

### Preparation of brain homogenates

Frozen brain tissue from frontal cortex (Brodmann Area 9) was dissected and approximately 0.1 mg of brain tissue is weighed out and added to 900 µl of TBS buffer (50 mM Tris-HCl, 175 mM NaCl, pH 7.4) with protease inhibitors (1X). Homogenization was done using the Precellys homogenization machine (Cryolys - Bertin) using their custom soft tissue homogenization CK14 tubes. Two cycles of 15 s homogenization were done at 5000 rpm with 5 min waiting time in between. Homogenate was centrifuged at 14,000 g, at 4 °C for 3 min. The supernatant from this was treated as the soluble TBS fraction used for the assays. The total protein concentration was measured for each sample using BCA protein assay kit (Pierce, Thermo Scientific). Samples were then diluted and aliquoted at a final concentration of 1 mg/ml. Aliquots were stored at −80 °C till use.

### Seeding amplification immunoassay (SAIA) for brain homogenates and CSF

For the seeding amplification immunoassay, 384 well plates (Nunc MaxiSorp, NUNC) were coated with anti-α-synuclein mAb 2A1 as capture antibody (at 0.5 µg/ml for BH analysis and 1 µg/ml for CSF analysis) in 0.2 M NaHCO3 and incubated overnight (ON) at 4 °C. After washing plate 3x (with PBS containing 0.05% tween-20; PBST) the plate was blocked for 2hrs at 37 °C (in PBST containing 2.5% gelatin) followed by a second wash. The brain homogenates were then added at a final total protein concentration of 5 µg/ml in 40 mM Phosphate buffer with 180 mM NaCl and 0.00375% SDS containing full length recombinant α-synuclein monomers (10 µg/ml) (50 µl/well). For analysis of CSF samples, CSF was diluted to 50% with equal volume of reaction buffer containing α-synuclein monomers (25 µg/ml) (50 µl/well). Following addition of samples in reaction buffer, plates are incubated at RT overnight(18hrs) or 4hrs, on plate shaker set to 450 rpm. After a third washing step, Biotinylated 2A1 was used as detection with 1 h incubation at 37 °C (at 0.1 µg/ml for BH analysis and 0.25 µg/ml for CSF analysis). Plates were then incubated in Streptavidin HRP (Jackson ImmunoResearch) at 1:5000 for 1 h at 37 °C. Plates were incubated with TMB substrate (Supersignal ELISA Femto, Pierce) in dark for 10 min. Reaction was stopped with 0.6N H2SO4 and the plate was read at 450 absorbance in the microplate reader (PerkinElmer).

### SAIA assay characterization: test of assay sensitivity

For the determination of minimal dilution of BH that can be detected by SAIA, dilutions of PDD, DLB and MSA brain homogenates were prepared and tested starting from a total protein concentration of 20 µg/ml (1:100 of the stock samples) followed by twelve 2x serial dilutions to reach a final dilution of 1:204k of the brain homogenate. Immediately prior to loading the samples, the prepared dilutions are added to the reaction buffer containing 10 µg/ml monomers.

Similarly, we tested to evaluate the SAIA sensitivity using recombinant preformed fibrils (PFFs). From a starting concentration of 5 ng/ml, PFFs were serially diluted 12 times until a final concentration of 0.05 ag/ml, and each dilution was spiked into reaction buffer containing 10 µg/ml α-synuclein monomers and control brain homogenates at 5 µg/ml or only 10 µg/ml α-synuclein monomers in artificial CSF.

### Seeding amplification assay (SAA) for brain homogenates and CSF

For brain homogenates, 160 μl of reaction mix composed of 40 mM phosphate buffer (pH 8.0), 1 mM citrate, 0.1 mg/mL recombinant monomeric αSyn (filtered through a 100 kD MWCO filter immediately before use) and 100 μM ThT was distributed in each well in a 96-well black plate with clear bottom (Nunc; Thermo Fisher, Waltham, MA) at a final volume of 200 μl per well. Reactions were performed in triplicates. For each test, we loaded 40 μl of BH of 0.1 mg/mL total protein concentration. The plate was then sealed with sealing tape and incubated in Omega FLUOstar plate reader (BMG Labtech) at 37 °C with intermittent shaking cycles: double orbital with a 1-min shake (500 rpm) and 15 min rest throughout the indicated incubation time. The sample was considered positive if two or more of the replicates were positive, otherwise the sample was classified as negative.

For CSF, wells were preloaded with 6 silica beads (Sigma-Aldrich, St Louis, MO), and 85 μl of a reaction mix prepared to give final reaction concentrations of 40 mM phosphate buffer (pH 8.0), 170 mM NaCl, 0.1 mg/mL recombinant monomeric αSyn (filtered through a 100 kD MWCO filter immediately before use), 100 μM ThT, and 0.0015% sodium dodecyl sulfate was distributed in each well. Then, 15 μl of CSF per sample was spiked in triplicates into corresponding wells. The plate was then sealed with a sealing tape and incubated in Omega FLUOstar plate reader (BMG Labtech) at 42°C with intermittent shaking cycles: double orbital with a 1-min shake (500 rpm) and 1-min rest throughout the indicated incubation time. For both protocols, ThT fluorescence readings were taken every 25 min with a bottom read using 450 ± 10 nm (excitation) and 480 ± 10 nm (emission) wavelengths. The sample was considered positive if 2 or more of the replicates were above the calculated threshold. The threshold was calculated as the average fluorescence for all samples within the first 10 h of incubation and 3 times the SDs.

### Statistical analysis

The data were deemed unsuitable for parametric analyses following tests of normality. Spearman's rank-order correlation coefficients were employed to explore correlations within the study cohorts. To assess distinctions between two diagnostic groups, the Mann–Whitney U test was employed. Meanwhile, the Kruskal-Wallis Test was applied for making comparisons across multiple groups. ROC Curve analysis was conducted independently for each cohort to evaluate diagnostic parameters of the SAIA. Based on these analyses, a common threshold for positivity was obtained using Youden Index calculation. The final threshold (1.7 ng/ml) was selected as the value that yielded a Youden index > 70% across all cohorts. This approach ensured that the threshold was data-driven yet consistent across cohorts. Data analysis and creation of the corresponding graphs were performed using GraphPad Prism 9 (GraphPad Software, Inc., San Diego, CA).

## Results

### Establishing proof-of-concept for SAIA using postmortem human brain and CSF samples

In this study, we utilized the in-house antibody 2A1 against soluble α-synuclein aggregates, which has been extensively characterized both here and in previous publications.^13^ This mouse IgG2a antibody specifically detects α-synuclein aggregates, as demonstrated by the filter retardation assay (Figure 2(a)), with minimal reactivity toward monomeric forms. It also detects fibrils prepared from truncated α-synuclein variants down to residue 122 (Figure 2(a)). Epitope mapping confirmed its binding to the C-terminal region (amino acids 113–123), while isotyping analysis determined it to be an IgG2a subclass antibody (Supplementary Figure 1).

A sandwich ELISA was developed using 2A1as the capture antibody and biotinylated-2A1 for detection, incorporating HNE-crosslinked α-synuclein oligomers as the standard calibrator. The assay displayed high linearity (R^2^ = 0.99; Figure 2(b)), a detailed protocol for HNE-α-synuclein oligomers preparation was recently published by our group.^54^ Monomeric α-synuclein was also tested in parallel and showed negligible signal, confirming the assay's specificity for α-synuclein oligomers (Figure 2(b)).

These results validate the assay's reliability in detecting α-synuclein aggregates with high specificity. The robustness of the assay was assessed by conducting repeat measurements of BH and CSF samples, as well as the calibrator standards, on three nonconsecutive days. Inter- and intra-assay variability were both observed to be less than 15% (Figure 2(c) and (d)).

Following a series of optimization steps, we demonstrated proof-of-concept of the SAIA technique in a small pilot study of *postmortem* BH samples. The BHs were derived from superior frontal cortex of individuals with clinico-pathologically-confirmed PDD and age-matched controls. The BHs were incubated for 18hrs at RT with continuous mixing (450 rpm) in a reaction buffer containing recombinant α-synuclein monomers (10 µg/ml) and compared to those in reaction buffer without α-synuclein monomers (Figure 2(f)). Samples incubated with the substrate, α-synuclein monomers, showed higher levels of seeded α-synuclein in PDD samples compared to control samples which showed negligible levels. In contrast, no seeded α-synuclein was detected in samples incubated without the presence of monomers (Figure 2(f)). This finding confirms the amplifications of α-synuclein seeds present in the PDD samples, specifically promoted by α-synuclein monomers acting as a substrate for the seeding reaction, thereby proving the concept of the SAIA.

We then explored SAIA application in CSF samples collected from living subjects using a pilot cohort of 20 PD subjects and 20 age-matched controls. Similar to BHs tested, CSF samples were incubated with and without the presence of α-synuclein monomers (10 µg/ml) substrate. Consistent with the results obtained from BH samples, we observed a significant increase in α-synuclein seeding activity specifically in PD CSF samples, as evidenced by markedly higher levels of seeded α-synuclein compared to controls, which exhibited minimal α-synuclein seeding (*p* < 0.001) (Figure 2(f)).

To further validate the results generated by SAIA, we performed a side-by-side comparison with the traditional SAA method. The SAA assay confirmed that PDD were SAA test-positive, while the control samples were negative (Figures 2(g)). These consistent results between SAIA and SAA underscore the reliability and accuracy of our novel assay in detecting disease-specific α-synuclein seeding activity in different biological matrices.

### Assay sensitivity and robustness evaluation of SAIA: definition of detection limits

**Figure 3.**
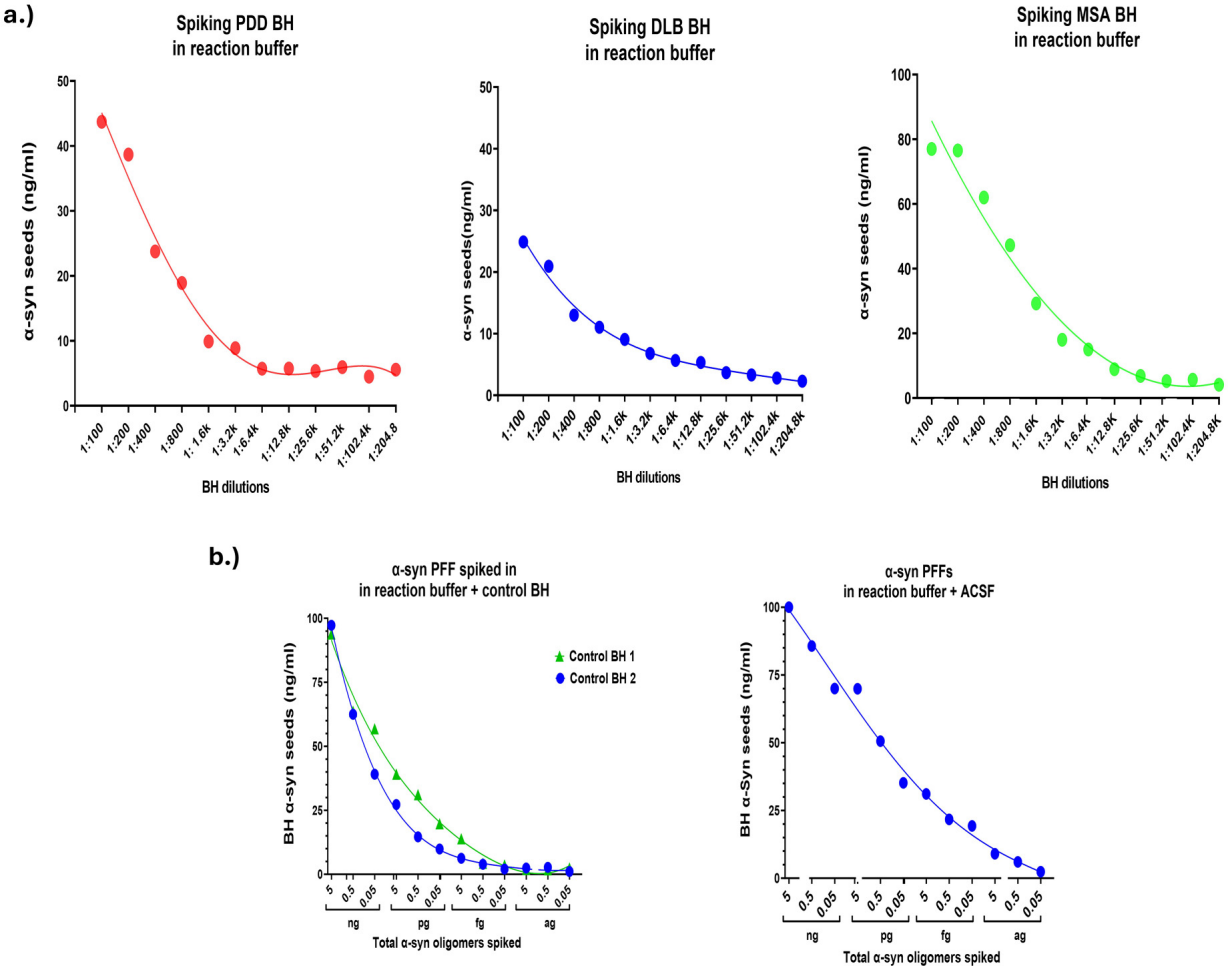
Characterisation of SAIA assay: sensitivity and robustness. (a) The panel illustrates the SAIA assay's sensitivity by determining the dilution, between 1:200 and 1:204.2k, of BH obtained from PD, DLB and MSA wherein seeded α-synuclein can be effectively detected. Sensitivity was established at approximate dilution of 1:6.4k of BH samples. (b) SAIA's detection capability is highlighted by assessing α-synuclein PFFs at series of dilutions from 5 nanograms to 0.05 attograms. Testing α-synuclein PFFs at different concentrations in control BH and artificial CSF (ACSF) showed sensitivity levels at 0.5 femtogram in BH and 0.5 attogram in ACSF buffer. *Error bars represent the standard error of the mean.*

Having confirmed that the SAIA concept works, we aimed to evaluate the assay's sensitivity and robustness. To assess the sensitivity of the assay and determine the minimum quantity of α-synuclein seeds that could be accurately amplified and quantified, we conducted comprehensive investigations using two distinct approaches. Firstly, we performed serial dilutions of BH derived from individuals with PDD, MSA, and DLB, spanning dilutions from 1:100 to 1:204,200. Subsequently, these diluted samples were incubated in a reaction buffer containing α-syn monomers as a substrate. The results encouragingly demonstrated the assay's remarkable sensitivity as it detected seeded α-synuclein in BH diluted up to 6400 times while being incubated only for a short period of time (18hrs) and with a very low concentration of the substrate (10 µg/ml) (Figure 3(a)), exhibiting a concentration-dependent pattern as evident in the decreasing levels of seeded α-synuclein in diluted samples. Secondly, we employed α-synuclein preformed fibrils (PFFs) in a series of quantities, ranging from 5 nanograms to 0.05 attograms, incubated for 18hrs with a reaction buffer containing α-synuclein monomers as a substrate. This incubation was performed using BH obtained from pathologically-confirmed controls or artificial CSF (ACSF). Remarkably, our assay successfully amplified and quantified as little as 0.5 attogram of α-synuclein PFFs in the ACSF buffer and 500 attograms in control BH samples (Figure 3(b)).

### Validating the SAIA assay: assessing CSF seeded α-synuclein levels in synucleinopathies and controls

**Figure 4.**
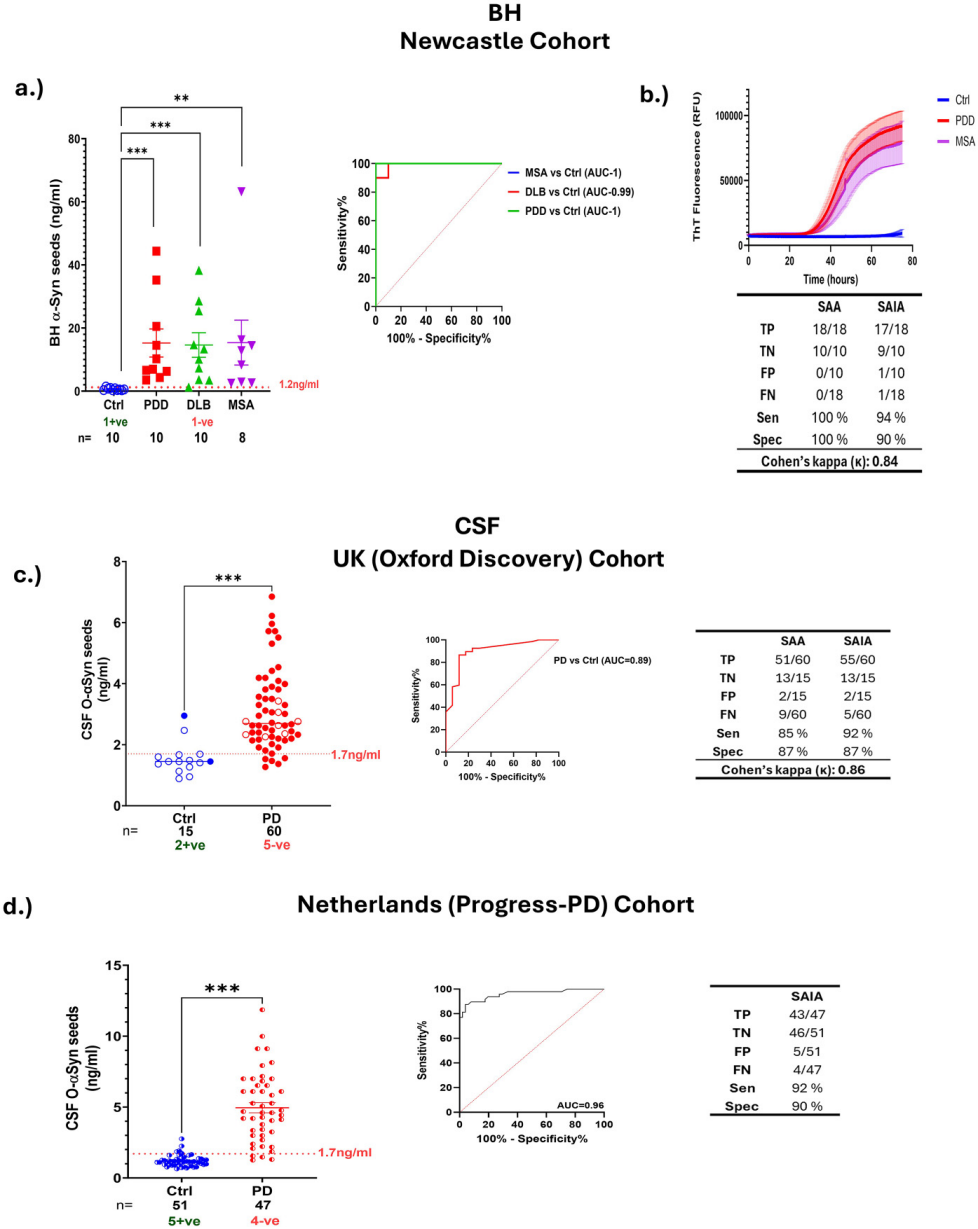
Validation of SAIA assay: BH and CSF analysis. (a) SAIA assay validated in a larger cohort of samples from brain homogenates from different synucleinopathies PDD, DLB, and MSA. Scatter plot and ROC analysis of measured seeded α-synuclein levels in BH samples of PDD, DLB, MSA vs control. (b) SAA reactions of BH samples seeded in triplicate from subjects confirmed with α-synuclein pathology (PDD, n = 10, MSA, n = 8) compared to controls (n = 9). The solid line represents the average ThT signal per group while error bars depict standard errors. (c, d.) These panels show a scatter plot depicting the measured levels of seeded α-synuclein in CSF samples from two cohorts: Netherlands (Progress-PD) cohort (n = 51 controls and 47 PD), and UK (Oxford Discovery) cohort (n = 15 controls and 60 PD). SAIA effectively distinguished PD cases from controls (*p* < 0.001). CSF samples with signals above the threshold (1.7 ng/ml red dashed horizontal line) calculated using Youden's Index of ROC analyses were considered positive while those below were negative. This outcome underscores SAIA's diagnostic potential as a robust tool for detecting PD. Open circles signify SAA-negative cases and closed colored circles illustrate SAA-positive cases. ROC curve analyses evaluate the diagnostic performance of SAIA in distinguishing between PD and control CSF samples and their corresponding sensitivities and specificities. Statistical comparisons between groups were calculated by Kruskal-Wallis Test and Mann-Whitney U test for two group comparison (***-p < 0.001, **-p < 0.01). Cut-off of 1.2 ng/ml for BH and 1.7 ng/ml for CSF samples was calculated from Youden's Index from ROC analysis. The error bars represent the standard error of the mean. Cohen's kappa was calculated to measure the degree of agreement between SAA and SAIA.

To validate the assay, we tested a larger cohort of *postmortem* BH samples, including those collected from subjects with clinic-pathologically confirmed PDD (n = 10), DLB (n = 10), MSA (n = 6 MSA-P and 2 MSA-C), and controls (n = 10); see Supplementary Table 1. We observed significantly higher levels of seeded α-synuclein in the synucleinopathies tested compared to controls. Using a threshold determined by ROC analysis to differentiate between positive and negative samples, we achieved a sensitivity of 100% and specificity of 90% for PDD, MSA, and 90% sensitivity and specificity for DLB samples against controls, with areas under the curve (AUC) values ranging from 0.99–1 (Figure 4(a); Supplementary Table 2). We explored the correlation between the levels of seeded α-synuclein measured by SAIA and the neuropathological load of α-synuclein, quantified as the percentage area of LBs stained in six areas of interest within the same region (superior frontal gyrus) assessed by SAIA, but from the contralateral hemisphere in PDD and DLB cases. Unfortunately, brain sections from the same region were not available for the MSA cases. When assessing PDD and DLB cases together, no significant correlation was found between the mean percentage area of LB staining and the levels of seeded α-synuclein. However, when analyzed separately, a significant positive correlation was observed in PDD cases (r = 0.75, *p* < 0.05), suggesting a relationship between LB burden and α-synuclein seeding activity in this group. In contrast, no correlation was detected in DLB cases (Supplementary Figure 2).

SAIA was validated for CSF samples by conducting a blind screening of two CSF cohorts: a Netherlands (Progress-PD) cohort (n = 47 PD cases and 51 controls) and a UK (Oxford Discovery) cohort (n = 60 PD cases and 15 controls). SAIA results indicated significantly higher levels of α-synuclein seeding activity in PD samples compared to controls (*p* < 0.001) (Figure 4(c) and (d); Supplementary Table 2). SAIA results of both cohorts showed clear distinction between PD and controls (*p* < 0.001) with sensitivities and specificities above 90% (UK Oxford Discovery cohort: 13/15 negative controls and 55/60 positive PD cases ; Netherlands cohort: 46/51 negative controls and 43/47 positive PD cases).

To compare SAA and SAIA diagnostic performance, we tested the BH samples using SAA (Figure 4(b)) and used previously published SAA results for CSF samples from the UK (Oxford Discovery) cohort.^26^ The BH results showed all SAA positive cases except one DLB case to also be SAIA positive and similarly 9 out of the 10 control negative cases were negative in both assays. A total of 12 PD cases and 1 control of this UK cohort presented inconsistent results between SAA and SAIA. Of the 12 PD cases, 8 showed positive SAIA results but negative SAA results, while the other 4 showed the opposite. The degree of agreement between SAA and SAIA was assessed using Cohen's kappa for both cohorts, yielding values of 0.84 for the BH cohort and 0.86 for the CSF cohort, indicating a high level of agreement. SAIA scatter plots in Figure 4(a) and (c) illustrate SAA-positive samples as closed colored circles and SAA-negative samples as open circles. These samples were repeated in SAIA to confirm the results. The Netherlands cohort was not tested in SAA. These results demonstrated high concordance between SAIA and SAA, confirming the reliability of SAIA in detecting disease-specific α-synuclein seeding activity.

### SAIA detection of seeded α-synuclein in idiopathic iRBD: implications for early detection

**Figure 5.**
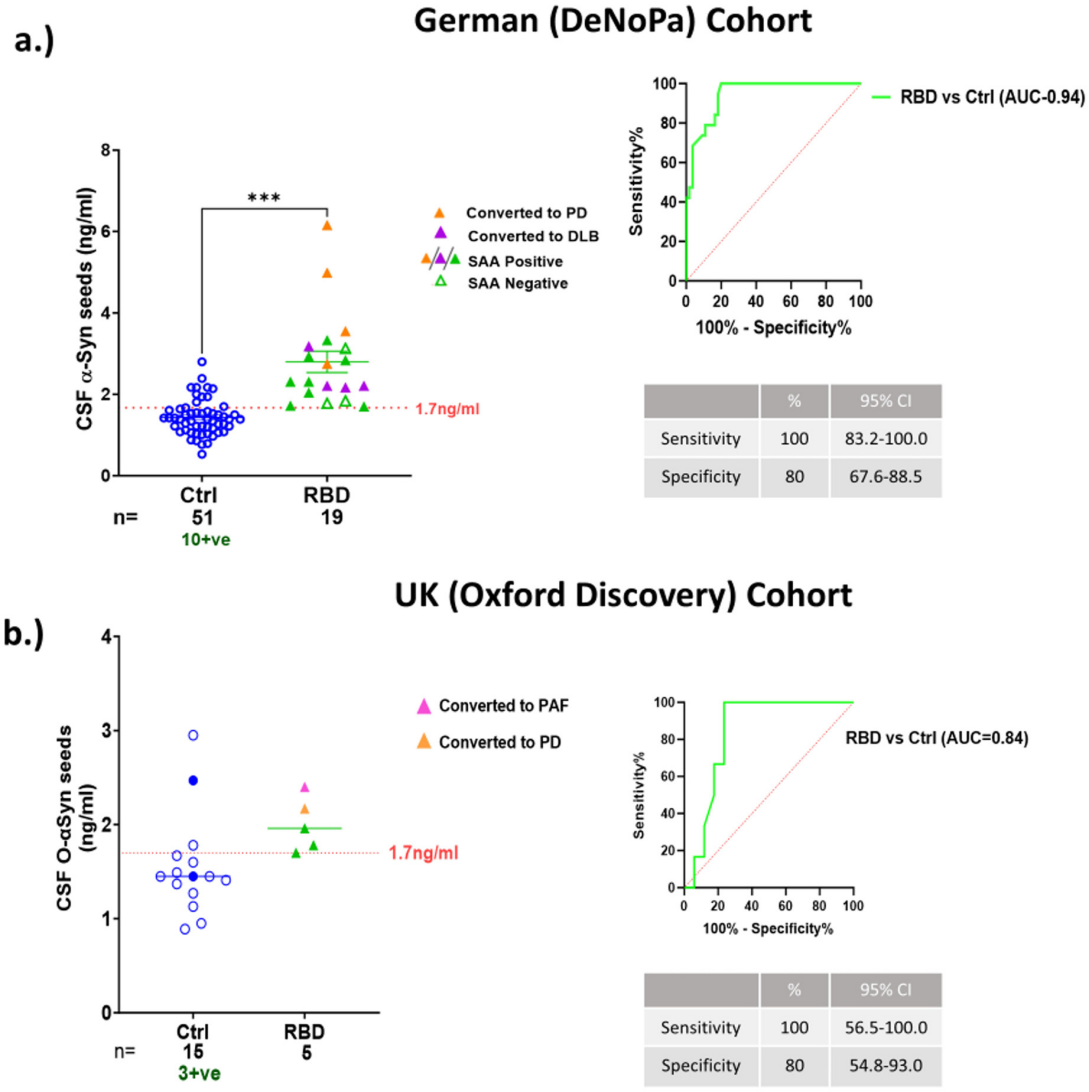
SAIA's detection sensitivity in prodromal synucleinopathies cases. SAIA's diagnostic performance in detecting prodromal α-synucleinopathies in patients with iRBD is highlighted. Analysis involved two cohorts (a) German (DeNoPa) cohort of 19 iRBD patients and 51 healthy controls, and (b) UK (Oxford Discovery) cohort of 5 iRBD patients and 15 controls. Scatter plots and ROC curve analyses underline SAIA's proficiency in quantifying the levels of seeded α-synuclein with a sensitivity of 100% and specificity of 80%. SAIA results and clinical longitudinal data were compared with SAA results and indicated in the graph as: open green triangles/circles- SAA negative samples, orange colored triangles- subjects that converted to PD, purple colored triangles- subjects that converted to DLB, and pink colored triangles-subjects that converted to PAF. Significant differences between groups were calculated by Man-Whitney test (***-p < 0.001, **-p < 0.01). Cut-off of 1.7 ng/ml was calculated from Youden's Index from ROC analysis. The error bars represent the standard error of the mean.

We tested a German cohort of patients with iRBD from the DeNoPa study comprising of 19 iRBD patients and 51 controls. Remarkably, SAIA was able to correctly identify all iRBD samples as test-positive (sensitivity = 100%, *p* < 0.001), while significantly discriminating them from controls (specificity = 80%, *p* < 0.001) (Figure 5; Supplementary Table 2). The same iRBD samples tested by our SAIA were previously tested in another study using SAA.^55^ Results from SAA study showed 3 of the 19 iRBD cases to be negative and highlighted in Figure 5 as open green triangles (also see Table 1). Two were among the lowest quantified samples, close to the cut-off value (1.7 ng/ml), using SAIA. An advantage presented in testing samples from the DeNoPa cohort is the availability of longitudinal clinical information up to 10yrs. This included information on the iRBD cases that had been converted to PD or DLB. The samples tested for this cohort by SAIA were obtained at baseline. Of the 19 iRBD cases tested from this cohort, four patients subsequently converted to DLB (represented as purple colored triangles), while four other iRBD cases converted to PD (represented as orange colored triangles) (Figure 5(a), Table 1). The levels of seeded α-synuclein detected in iRBD cases that converted to DLB were lower compared to those that converted to PD. The UK cohort included in this study contained 5 cases of iRBD with CSF samples which we also tested. Similar to the German iRBD cohort, all 5 iRBD cases tested positive in SAIA and corresponded as positive by SAA in previously published results (Figure 5(b), Table 1).^26^ Of the 5 iRBD cases, one case later converted to PD and another case converted to pure autonomic failure (PAF). These collective results suggest the reliability of SAIA as a potential early detection method for prodromal α-synucleinopathies.

**Table 1.** Comparison of SAA vs SAIA results in iRBD samples.

German cohort (DeNoPa)					
Case	SAA results (Positive/Negative)	SAIA results (Conc. ng/ml)	Final diagnosis	Comment	
iRBD 1	Positive	2.04	Dementia with Lewy bodies	
iRBD 2	Positive	2.21	Dementia with Lewy bodies	
iRBD 3	Positive	3.18	Dementia with Lewy bodies	
iRBD 4	Positive	2.17	Dementia with Lewy bodies	
iRBD 5	Positive	2.21	No conversion yet		
iRBD 6	Negative	1.84	No conversion yet	drop-out of RBS study: iRBD not certain	
iRBD 7	Positive	3.33	No conversion yet		
iRBD 8	Negative	1.78	No conversion yet	drop-out of iRBD study: PSG interpretation impaired due to severe sleep apnea	
iRBD 9	Negative	3.13	No conversion yet		
iRBD 10	Positive	2.93	No conversion yet		
iRBD 11	Positive	2.31	No conversion yet		
iRBD 12	Positive	2.84	No conversion yet		
iRBD 13	Positive	1.80	No conversion yet		
iRBD 14	Positive	2.31	No conversion yet		
iRBD 15	Positive	2.01	No conversion yet		
iRBD 16	Positive	4.99	Parkinson's Disease		
iRBD 17	Positive	6.16	Parkinson's Disease		
iRBD 18	Positive	3.55	Parkinson's Disease		
iRBD 19	Positive	2.75	Parkinson's Disease		
UK Cohort (Oxford Discovery)					
iRBD 1	Positive	2.17	Parkinson's Disease		
iRBD 2	Positive	2.40	Pure autonomic failure	
iRBD 3	Positive	1.96	No conversion yet		
iRBD 4	Positive	1.78	No conversion yet		
iRBD 5	Positive	1.70	No conversion yet		

A select number of positive, negative, and all borderline cases from each group were retested to confirm the results obtained. Retest results confirmed original results obtained. We were unable to retest the control samples due to lack of sample availability.

### Evaluating SAIA specificity in distinguishing synucleinopathies from non-synucleinopathy disorders

**Figure 6.**
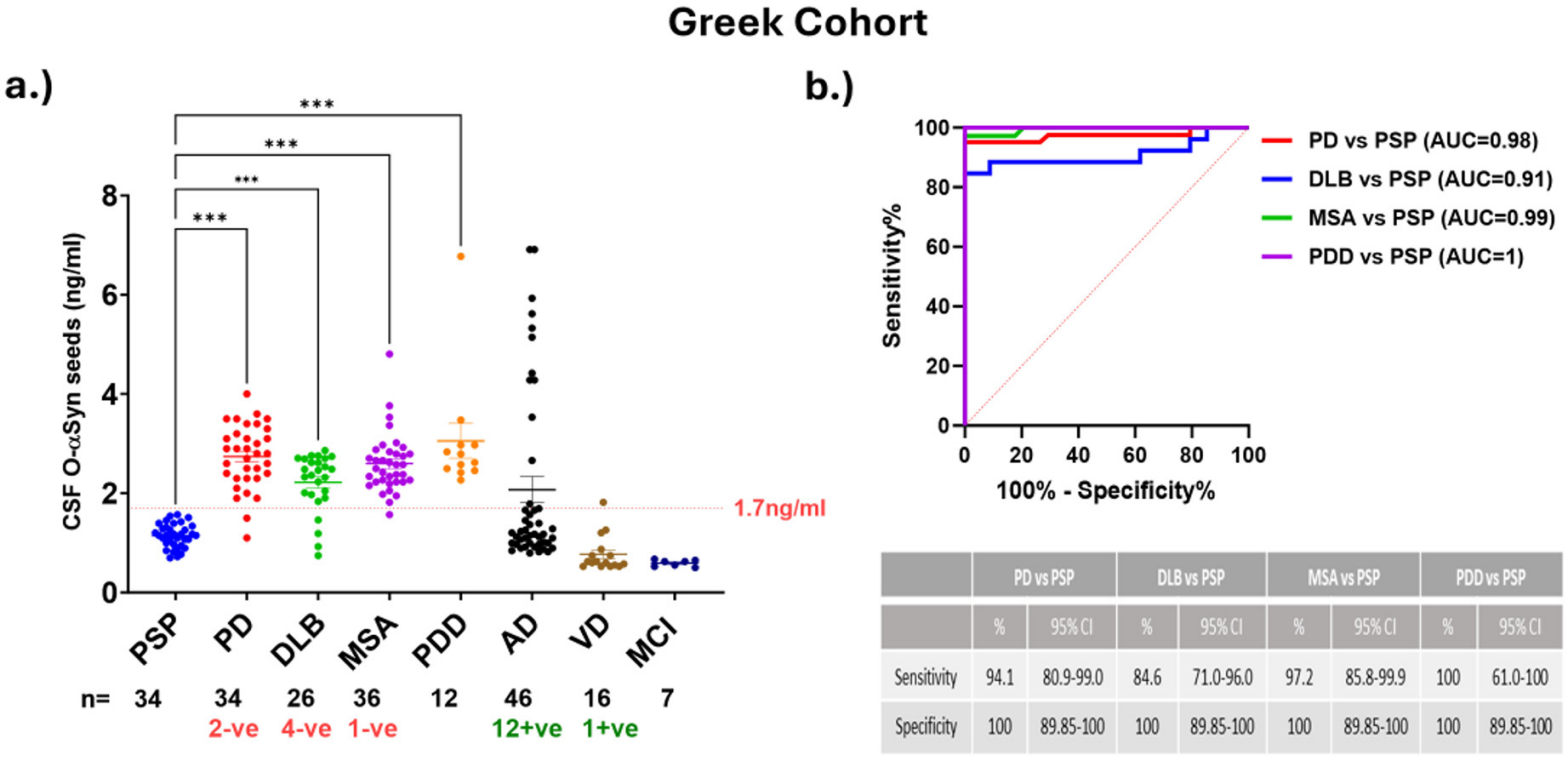
SAIA specificity in detecting synucleinopathies. (a) This figure displays SAIA's specificity in detecting synucleinopathies. CSF samples from PD, PDD, DLB, and MSA patients (n = 41, 6, 26, and 36) are compared with non-synucleinopathy patients with PSP, AD, VaD, or MCI (n = 34, 46, 16, and 7). (b) ROC curve demonstrating SAIA's ability to differentiate between PSP, as a control group, and the synucleinopathy groups PD (red line; Sensitivity=94%), DLB (blue line; Sensitivity = 85%), MSA (green line; Sensitivity=97%), PDD (purple line; Sensitivity = 100%). Significant differences between groups were calculated by Kruskal- Wallis Test (***- p *<* 0.001, **-p *<* *0.01). Cut-off of 1.7* *ng/ml was calculated from Youden's Index from ROC analysis. The error bars represent the standard error of the mean*.

To assess the specificity of SAIA for synucleinopathies, we analyzed CSF samples from a Greek cohort consisting of PD, PDD, DLB, and MSA patients (n = 34, 12, 26, and 36, respectively), as well as non-synucleinopathy including PSP, AD, VaD, and MCI patients (n = 34, 46, 16, and 7, respectively- Supplementary Table 1). With the exception of 12 out of 46 (26%) AD cases and 1 VaD case that tested as “positive”, the majority of non-synucleinopathy cases showed a negative result for seeded α-synuclein. Collectively our results demonstrated the high specificity of SAIA for synucleinopathies, with sensitivities ranging from 84% to 100% and specificities of 100% in relation to tauopathy PSP. While significant differences were not observed between the synucleinopathies tested, a trend of lower levels of seeded α-synuclein was observed in DLB cases (mean-2.2 ± 0.6) compared to PD (mean = 2.7 ± 0.6), PDD (mean = 3.1 ± 1.2), and MSA (mean = 2.6 ± 0.6). The AUC values were 0.98 for PD, 1 for PDD, 0.99 for MSA, and 0.91 for DLB, further supporting the discriminative power of SAIA (Figure 6(a) and (b); Supplementary Table 2).

### Improving SAIA specificity for distinguishing between synucleinopathies

Under standard SAIA conditions (18-h incubation), SAIA separated synucleinopathies from non-synucleinopathy disorders but did not significantly differentiate specific synucleinopathies (e.g., PD vs MSA). This was in contrast to previous studies using SAA, which demonstrated the ability to differentiate these groups based on distinct aggregation kinetics. To address this limitation, we optimized SAIA by performing timepoint analyses to determine whether varying incubation times could enhance the assay's specificity for distinguishing between PD and MSA.

**Figure 7.**
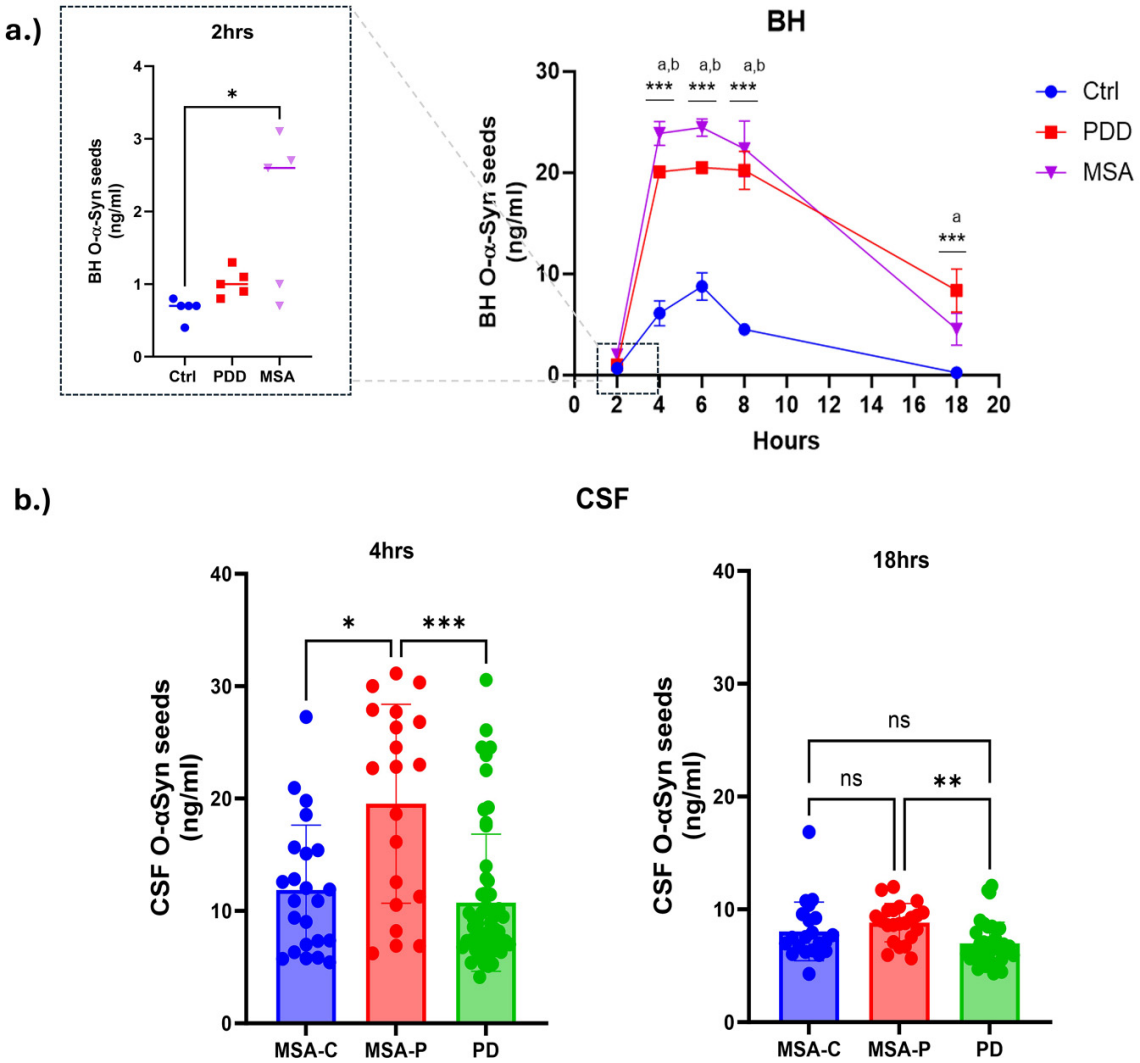
Timepoint optimization improves SAIA specificity for distinguishing synucleinopathies. (a) Aggregation kinetics of α-synuclein seeds in brain homogenate (BH) samples from MSA, PD, and control patients at multiple incubation timepoints (2, 4, 6, 8, and 18 h). MSA cases (n = 5) show significant early aggregation compared to controls (n = 5) at 2 h (*p* < 0.05), with PD cases (n = 5) trending higher but not reaching significance. (b) Comparison of α-synuclein seed levels in CSF samples from MSA-C, MSA-P, and PD cases (n = 23, 20, and 57, respectively) at 4 h. MSA-P shows significantly higher levels of seeded α-synuclein compared to MSA-C and PD (*p* < 0.05 and 0.001 respectively). (c) Comparison of α-synuclein seed levels in CSF samples from a smaller Netherlands (Progress-PD) cohort (Ctrl: n = 8; PD: n = 9) at 4 and 18 h. At 4 h, PD cases show significantly higher levels of α-synuclein seeds compared to controls (*p* < 0.001), a difference that remains robust at 18 h (*p* < 0.001). Significant differences between groups were calculated by Mann-Whitney or Kruskal-Wallis test (*-p *<* 0.01, **-p *<* 0.05, ***-p *<* *0.001; a-p* *<* *0.001 PDD vs Ctrl, b-p* *<* *0.001 MSA vs Ctrl). Error bars represent the standard error of the mean*.

A series of timepoint experiments were conducted using BH samples from MSA and PD patients, with incubations at 2, 4, 6, 8, 14, and 18 h. MSA cases exhibited rapid aggregation, with detectable seeded α-synuclein as early as 2 h (MSA mean = 2.02 ± 1.09 vs Ctrl mean = 0.66 ± 0.15, *p* < 0.05), while PD cases showed a trend towards higher levels (mean = 1.02 ± 0.19, *p* = 0.12). By the 4 and 6-h timepoints, significant differences were observed between both MSA and PD compared to controls, with MSA showing a higher trend than PD. By 8 h, levels began to drop, with a more pronounced reduction at 18 h, especially in MSA cases (Figure 7(a)). These findings suggest that prolonged incubation (18 h) may obscure kinetic differences between MSA and PD, leading to an overlap in α-synuclein seed detection.

To further explore this, we conducted timepoint analyses using CSF samples from a UK cohort (UCL) consisting of MSA (n = 43) and PD (n = 57) cases. The MSA group included MSA-C (n = 23) and MSA-P (n = 20) cases. Incubating sample for 4hrs instead of 18hrs showed that MSA-P samples (mean = 19.54 ± 8.9) had significantly higher levels of α-synuclein seeds compared to MSA-C (mean = 11.88 ± 5.76, *p* < 0.05) and PD cases (mean = 10.74 ± 6.1, *p* < 0.001), suggesting that shorter incubation times enhance SAIA's specificity for distinguishing MSA from PD (Figure 7(b)).

Given that the earlier cohorts were tested with the longer incubation protocol, we sought to investigate how shorter incubation would affect the results previously achieved for PD. Due to sample availability restriction, we could only test a small number of the Netherlands (Progress-PD) cohort (Ctrl: n = 8; PD: n = 9). At 4 h, PD cases showed significantly higher levels of α-synuclein seeds compared to controls (*p* < 0.001), with a clear separation between the groups. This separation was maintained at 18 h, where PD cases continued to show significantly elevated α-synuclein levels compared to controls (*p* < 0.001) (Supplementary Figure 3). Unlike the overlap observed in disease-specific comparisons (e.g., MSA vs PD), these results reinforce the robustness of SAIA for distinguishing PD from controls at both timepoints.

## Discussion

Early and accurate detection of α-synuclein pathology is paramount for effective disease management and intervention. SAA, an emerging technique over recent years, has shown considerable promise as a powerful tool to detect misfolded forms of α-synuclein. Studies to date have demonstrated high diagnostic sensitivities (exceeding 90%) and specificities ranging of 82–100% across various sample types.^15,19,56^ SAA limitations that include its binary output, protocol variability, and technical complexity have hindered its widespread adoption. This study addresses these gaps by combining seeding-based amplification with quantitative detection, offering a more accessible and versatile approach that may improve clinical implementation.

This study presents the first comprehensive validation of SAIA in both postmortem brain and CSF samples across multiple independent cohorts. Initial pilot data confirmed the assay's ability to detect α-synuclein seeds in disease cases while remaining negative in controls. These early findings laid the foundation for expanded validation in larger, clinically diverse populations. Sensitivity assessments demonstrated that SAIA could detect attogram-level quantities of seeds with short incubation times (∼18 h) and low monomer concentrations (∼10 µg/ml), highlighting its efficiency compared to SAA protocols, which typically require higher substrate concentration (10x–100x more), longer incubation (3–7days), and more complex setups and variations of protocols for different biofluids (addition of beads, detergents, etc.).^57,58^ Performance metrics were robust with inter- and intra-assay variability below 15%. Notably, SAIA's simplified workflow does not require specialized instrumentation, making it suitable for broad laboratory adoption.

SAIA showed consistent performance in larger cohorts of postmortem brain and CSF samples, accurately distinguishing synucleinopathy cases from controls with high sensitivity and specificity (above 90% and 85% respectively). To further assess the clinical utility of SAIA, we performed a direct comparison with the established SAA method. While direct comparisons with SAA revealed strong concordance, some discordant cases were identified, with majority of these samples being detected positive for α-synuclein seeds by SAIA while tested negative by SAA. These findings suggest that SAIA may capture forms of α-synuclein not readily detected by ThT fluorescence, likely due to differences in structural properties. This highlights a key strength of SAIA in detecting early or structurally distinct seeds that may otherwise be missed.

The assay's ability to detect seeded α-synuclein in prodromal cases, such as iRBD, further supports its value for early diagnosis. Longitudinal clinical data for these cases provided additional insight into disease progression. All iRBD cases tested were SAIA-positive, including those that later converted to PD, DLB, or PAF. These results align with those previously published using SAA.^16,25,26,59^ Notably, higher seed levels were observed in iRBD cases that converted to PD compared to those that converted to DLB, consistent with trends in the Greek cohort where DLB cases often exhibited lower SAIA signals. These observations suggest potential differences in aggregation dynamics across synucleinopathy subtypes. Such findings also raise the possibility that quantitative measures from SAIA may serve as an early diagnostic tool for prodromal α-synucleinopathies. However, we acknowledge that further validation through larger longitudinal studies would be invaluable in fully establishing its clinical utility in this context.

SAIA also detected α-synuclein seeds in a subset of AD cases, consistent with previous reports of co-pathology and prior findings using SAA.^8,16,25,42,60–64^ Interestingly, some of these AD cases showed higher seed levels than those observed in PD or DLB, which may reflect differences in the structural properties of the aggregates.^65^ Notably, no α-synuclein pathology was detected in MCI cases, suggesting that such pathology may emerge later in the disease course, in line with autopsy studies showing α-synuclein aggregation appears in more advanced stages of AD.^66^ The comparatively low detection rate (26.6%) in our AD cohort may thus reflect early-stage disease or low pathology burden. Additionally, as SAIA targets soluble aggregates, it may capture species not detected by assays focused on insoluble fibrils like Lewy bodies. This difference in detection may also account for lower levels observed in DLB relative to some AD cases. These explanations, however, remain speculative and will require validation in future studies.

SAIA could not significantly differentiate between these distinct synucleinopathies tested in this study, however a trend of lower levels of seeded α-synuclein in DLB cases compared to PD, PDD, and MSA was observed. Several studies have demonstrated that there are differences in the strains of α-synuclein present in different α-synucleinopathies with some cases also indicating heterogeneity of species found.^56,67–70^ Nonetheless SAIA technique holds potential for enhanced sensitivity to better stratify and distinguish between these diseases through the use of other strain-specific anti-α-synuclein antibodies, different reaction buffer,^24,56^ truncated α-synuclein as substrate^71^ or even possibility exploring combining SAIA results with other methods, such as autonomic function testing, neuroimaging, and/or genetics.^72–74^

To explore whether aggregation kinetics could help distinguish MSA from PD as seen in SAA, we tested shorter incubation times. In brain homogenates, MSA cases aggregated earlier than PD, with seeds detectable at 2 h and peaking before 8 h. PD samples reached peak signal later. By 18 h, signals in both declined, particularly in MSA, leading to overlap and masking these kinetic differences possibly due to the shift toward insoluble aggregates less detectable. Additionally, it is possible that extended shaking may contribute to partial dissociation of the antibody from the aggregates, further reducing the detectable signal. In CSF, a similar pattern emerged. At 4 h, MSA-P cases showed significantly higher seed levels than MSA-C and PD, supporting distinct kinetics between MSA subtypes. These results align with findings by Bargar et al.,^75^ who reported low seeding activity in MSA-C compared to MSA-P. While our main goal was to differentiate MSA from PD, these observations suggest that early-phase kinetics may reflect underlying strain differences, better captured at shorter timepoints.

To confirm the diagnostic utility of shorter incubation, we reanalyzed a subset of CSF samples from the Netherlands cohort. PD remained clearly separable from controls at 4 h, with robust signal maintained at 18 h, indicating that both timepoints support diagnostic accuracy. However, shorter incubation may offer advantages for distinguishing subtypes like MSA-P and PD. Collectively, these findings highlight the flexibility of SAIA to be tailored for specific diagnostic objectives whether enhancing sensitivity through longer incubation or achieving better disease stratification through early aggregation timing. Further studies across additional cohorts and incubation parameters will be critical to validate and expand these findings.

We acknowledge several limitations. First, the retrospective determination of the positivity threshold. Future studies employing predefined cutoffs in independent cohorts will be critical for confirming the diagnostic utility of this assay. Second, lower specificity rate found in the iRBD cohort (∼80%), which may reflect incidental Lewy body pathology in controls. The assay's high sensitivity may allow detection of subclinical or early-stage pathology, as supported by autopsy data showing incidental Lewy body disease in aging populations (range 40–100 years).^76^ Third, during assay optimization, we also observed that SAIA performance can be more sensitive to preanalytical factors such as freeze-thaw cycles and batch-to-batch variation in substrate preparations. These sources of variability highlight the need for further technical refinements and standardization to enhance the assay's robustness and reproducibility. Finally, future longitudinal studies are essential to validate these interpretations, evaluate SAIA in target engagement monitoring, and further refine assay parameters for clinical use.

In summary, we have developed a quantitative high throughput immunoassay for synucleinopathies with high diagnostic potential for use in diagnostics, longitudinal studies, and clinical trials. SAIA represents a significant methodological advance, simultaneously amplifying and quantifying α-synuclein seeds, offering a streamlined approach for diagnosing, monitoring, and assessing drug engagement in synucleinopathies. The assay's high sensitivity, specificity, and versatility make it a promising tool for the detection, quantification, and monitoring α-synuclein pathology *in vivo*. Importantly, this simplified assay can be readily adopted in most clinical and research laboratories, as it relies on a traditional colorimetric ELISA platform. This eliminates the need for sophisticated and costly equipment or specialized laboratories requiring extensive technical expertise.

## Supplemental Material

sj-xlsx-1-pkn-10.1177_1877718X251379292 - Supplemental material for Quantitative measurements of α-synuclein seeds in CSF inform diagnosis of synucleinopathies Supplemental material, sj-xlsx-1-pkn-10.1177_1877718X251379292 for Quantitative measurements of α-synuclein seeds in CSF inform diagnosis of synucleinopathies by Ilham Y Abdi, Indulekha P Sudhakaran, Simona S Ghanem, Nishant N Vaikath, Nour Majbour, Yee Y Goh, Nirosen Vijiaratnam, Christine Girges, Vasilios C Constantinides, Elisabeth Kapaki, George P Paraskevas, Sandrina Weber, Gholam Adeli, Kostas Vekrellis, Daniel Erskine, Michele Hu, Thomas Foltynie, Henry Houlden, Laura Parkkinen, Wilma DJ van de Berg, Brit Mollenhauer, Michael G Schlossmacher, and Omar MA El-Agnaf in Journal of Parkinson's Disease

sj-docx-2-pkn-10.1177_1877718X251379292 - Supplemental material for Quantitative measurements of α-synuclein seeds in CSF inform diagnosis of synucleinopathies Supplemental material, sj-docx-2-pkn-10.1177_1877718X251379292 for Quantitative measurements of α-synuclein seeds in CSF inform diagnosis of synucleinopathies by Ilham Y Abdi, Indulekha P Sudhakaran, Simona S Ghanem, Nishant N Vaikath, Nour Majbour, Yee Y Goh, Nirosen Vijiaratnam, Christine Girges, Vasilios C Constantinides, Elisabeth Kapaki, George P Paraskevas, Sandrina Weber, Gholam Adeli, Kostas Vekrellis, Daniel Erskine, Michele Hu, Thomas Foltynie, Henry Houlden, Laura Parkkinen, Wilma DJ van de Berg, Brit Mollenhauer, Michael G Schlossmacher, and Omar MA El-Agnaf in Journal of Parkinson's Disease
