# Supplementary material for: Quantitative measurements of α-synuclein seeds in CSF inform diagnosis of synucleinopathies
Source: J Parkinsons Dis. 2025 Oct 3;15(8):1412–30. doi: 10.1177/1877718X251379292 (PMC13347542; doi:10.1177/1877718X251379292)
Supplement: sj-docx-2-pkn-10.1177_1877718X251379292 - Supplemental material for Quantitative measurements of α-synuclein seeds in CSF inform diagnosis of synucleinopathies [file sj-docx-2-pkn-10.1177_1877718X251379292.docx]

**Supplementary Figure 1.**


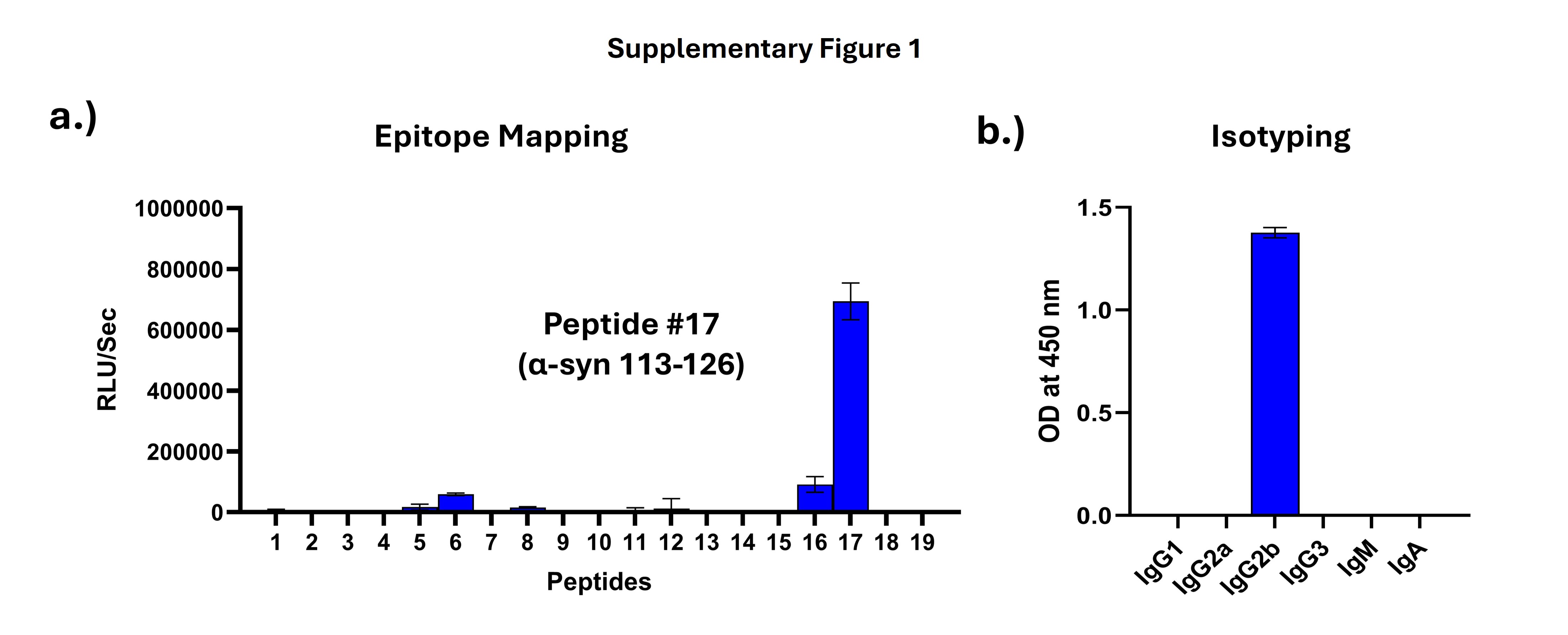


**Epitope mapping and isotyping of 2A1 antibody**

1. Epitope mapping analysis using α-synuclein peptides, demonstrating that 2A1 specifically binds to peptide 17, corresponding to amino acids 113–123 of α-synuclein. (b) Isotyping analysis confirming that 2A1 is an IgG2a subclass antibody with a kappa light chain.


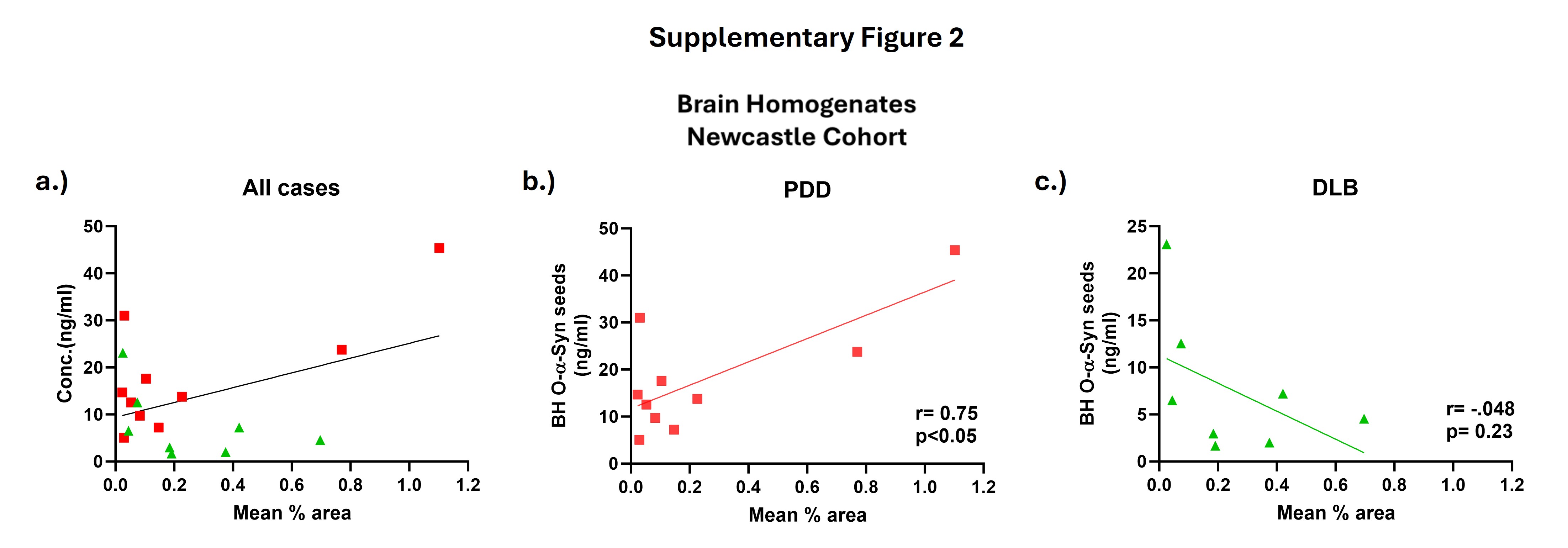


**Supplementary Figure 2.**

**Correlation between α-synuclein seeding activity and Lewy body burden in brain homogenates from the Newcastle cohort.** Scatter plots show the relationship between α-synuclein seeding activity measured by SAIA and the mean percentage area of Lewy body (LB) staining in **(a)** all cases combined (PDD and DLB), **(b)** PDD cases alone, and **(c)** DLB cases alone. A significant positive correlation is observed in PDD cases (b, red squares, r = 0.75, p < 0.05), while no correlation is detected in DLB cases (c, green triangles, r = -0.30, p = 0.40). Linear regression lines are shown for each group.


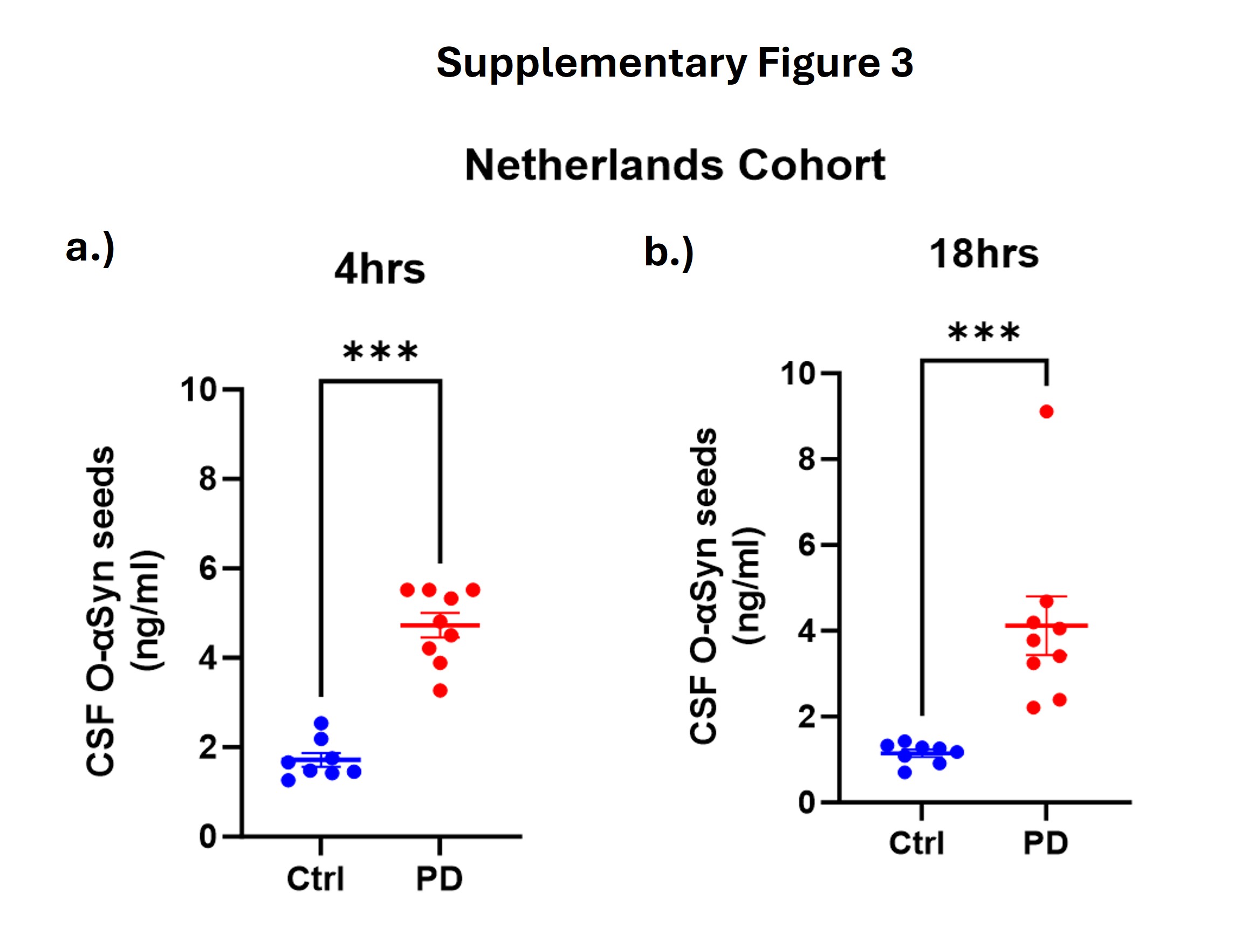


**Supplementary Figure 3.**

**SAIA distinguishes PD from controls in the Netherlands (Progress-PD) cohort at both 4 and 18 hours**

Comparison of α-synuclein seed levels in CSF samples from PD (n=9) and control (n=8) subjects at 4 hours (a) and 18 hours (b). At both timepoints PD cases show significantly higher levels of seeded α-synuclein compared to controls (p < 0.001).

*Significant differences between groups were calculated by the Mann-Whitney test (***-p<0.001). Error bars represent the standard error of the mean.*
